# Using unique ORFan genes as strain-specific identifiers for *Escherichia coli*

**DOI:** 10.1186/s12866-022-02508-y

**Published:** 2022-05-18

**Authors:** Marta Ferrandis-Vila, Sumeet K. Tiwari, Svenja Mamerow, Torsten Semmler, HECTOR consortium, Marta Ferrandis-Vila, Sumeet K. Tiwari, Boas van der Putten, Nguyen V. Trung, Rik Oldenkamp, Martin Bootsma, Sebastien Matamoros, Hoa T. Ngo, Julio Alvarez, Jennifer M. Ritchie, Amanda Fivian-Hughes, Angelika Fruth, Joy Leng, Roberto M. La Ragione, Maria Ugarte-Ruiz, Astrid Bethe, Stefan Schwarz, Torsten Semmler, Constance Schultsz, Christian Menge, Christian Berens, Christian Menge, Christian Berens

**Affiliations:** 1 Friedrich-Loeffler-Institut – Federal Research Institute for Animal Health, Institute of Molecular Pathogenesis, Naumburger Straße 96a, 07743 Jena, Germany; 2 Microbial Genomics, Robert Koch Institute, Berlin, Germany

**Keywords:** ORFan gene, Strain identification, Multiplex PCR, qPCR, *E. coli*, Host-specificity, Animal experiment

## Abstract

**Background:**

Bacterial identification at the strain level is a much-needed, but arduous and challenging task. This study aimed to develop a method for identifying and differentiating individual strains among multiple strains of the same bacterial species. The set used for testing the method consisted of 17 *Escherichia coli* strains picked from a collection of strains isolated in Germany, Spain, the United Kingdom and Vietnam from humans, cattle, swine, wild boars, and chickens. We targeted unique or rare ORFan genes to address the problem of selective and specific strain identification. These ORFan genes, exclusive to each strain, served as templates for developing strain-specific primers.

**Results:**

Most of the experimental strains (14 out of 17) possessed unique ORFan genes that were used to develop strain-specific primers. The remaining three strains were identified by combining a PCR for a rare gene with a selection step for isolating the experimental strains. Multiplex PCR allowed the successful identification of the strains both in vitro in spiked faecal material in addition to in vivo after experimental infections of pigs and recovery of bacteria from faecal material. In addition, primers for qPCR were also developed and quantitative readout from faecal samples after experimental infection was also possible.

**Conclusions:**

The method described in this manuscript using strain-specific unique genes to identify single strains in a mixture of strains proved itself efficient and reliable in detecting and following individual strains both in vitro and in vivo, representing a fast and inexpensive alternative to more costly methods.

**Supplementary Information:**

The online version contains supplementary material available at 10.1186/s12866-022-02508-y.

## Background

The tracing of microbes in complex biological systems is indispensable to answer many scientific questions in applied, clinical, and environmental microbiology. Bacterial identification at the strain level is highly challenging since closely related bacteria may have similar morphologic and physiologic profiles, and strains belonging to the same bacterial species and even to the same bacterial family, are indistinguishable solely by morphological methods [1]. Techniques to identify strains have evolved and advanced in the past decades, but all available methods have limitations and flaws, and none of them is 100% reliable or accurate [2]. Differentiation of bacterial strains based on their genome information has become the preferred approach due to its excellent resolution, high reliability, and easy availability [3]. Genotyping methods can be grouped into three major categories [3]. The first is based on DNA fragment patterns, in which amplification and/or enzymatic digestion of bacterial DNA is followed by electrophoretic resolution of differently sized fragments, the pattern of which serves as specific species or strain identifier. For amplicon-based classification, the DNA sequence of a reference genome has to be at least partially known. Second, DNA-hybridisation systems deploy nucleic acid-based probes, labelled fragments of known sequence complementary to their corresponding targets, which are detected after probe binding. Third, DNA sequence-based genotyping, the most powerful tool currently being used to classify bacteria, utilizes strain-specific variations, such as single nucleotide polymorphisms (SNP) [4] as well as deletion or addition of genetic material [5, 6]. These methods all rely on specific differences between individual strains, which have to be at least qualitatively, but preferably quantitatively, detectable within the context of a complex microbial environment, in which one or more strains of the target species may already be present.

*Escherichia coli* is a commensal member of the vertebrate gut, but certain strains, grouped into *E. coli* pathovars, have acquired virulence genes, mainly located on mobile elements, on multiple occasions resulting in a high degree of genomic flux [7, 8]. *E. coli* has an open *pan*-genome, i.e., the number of genes it contains (consisting of core and accessory genes) increases with the number of additional genomes sequenced. Many accessory genes are only present in one strain (also known as singletons) or in a few strains [9, 10]. Such genes with no known relatives or homologues in species belonging to other lineages are known as orphan or ORFan genes [11]. Improved genotyping and sequencing techniques during the last two decades have led to the discovery of large numbers of ORFan genes present within bacterial genomes [11, 12].

New studies analysing the genomes of pathogenic and non-pathogenic bacteria have shown that all genomes of a single species have their own share of specific and unique ORFan genes, which are common to some strains but are not found consistently in all members of the species: this part of the genome is also considered to belong to the “variable or accessory genome” [13–15]. Several ORFan genes are lineage-specific, while others can even be strain-specific and seem to contribute to a particular strain’s characteristics, including potential pathogenicity [16, 17]. These features qualify specific ORFan genes to serve as molecular tracer for bacteria at the strain level. In this study, we describe an ORFan gene-based identification method characterized by the generation and development of PCR primers specific at the bacterial strain level. An extensive *E. coli* library containing 1198 whole-genome-sequenced strains collected by the consortium served as database to identify suitable ORFan genes. Specific regions within these ORFan genes were tested against larger sequence databases to increase the marker’s probability of being restricted to a single strain. Specific PCR and quantitative PCR (qPCR) primers derived from the search results were successfully applied in vitro and in vivo in an animal infection experiment.

## Materials and methods

### Strain selection

One thousand one hundred and ninety-eight *E. coli* strains were provided by all HECTOR project partners and whole-genome-sequenced using short-read technology (van der Putten, B., Tiwari, S.K., HECTOR consortium, Semmler, T. and Schultsz, C., unpublished data 10.1101/2022.02.08.479532). The sequencing was executed using Illumina MiSeq (2 × 150 bp, 2 × 250 bp, 2 × 300 bp paired reads) and the Illumina HiSeq 4000 system (2 × 150 bp paired reads). As quality control, adapter sequences and low-quality bases within raw reads were trimmed. For genome assembly and annotation, adapter-trimmed reads were assembled with SPAdes v3.13.1 using read correction [18]. Scaffolds smaller than 500 bp were discarded. QUAST v5.0.0 was used to assess assembly quality using default parameters [19]. Data on the assemblies is given in Supplementary Table S1. Seventeen strains, carrying extended-spectrum beta-lactamase (ESBL) genes, were selected from this collection for an animal study to assess their colonization properties in livestock animals in parallel with an infection approach using a mixture of strains (cocktail) (Table 1).

**Table 1:** *E. coli* strains used in this study (*n* = 17)

Strain name	Strain ID	Host	Country of origin
**ChH1**	21225_2#112	Chicken	Vietnam
**ChH2**	SAP1847	Human	UK
**ChH3**	SAP1710	Human	UK
**C1**	IMT38565	Cattle	Germany
**C2**	R45	Cattle	Germany
**C3**	IMT13936	Cattle	Germany
**C4**	IMT34414	Cattle	Germany
**C5**	IMT10909	Cattle	Germany
**C6**	9475_4#43	Cattle	Germany
**P1**	IMT39234	Pig	Germany
**P2**	IMT28138	Pig	Germany
**P3**	39533	Pig	Germany
**P4**	IMT38723	Pig	Germany
**P5**	IMT38701	Pig	Germany
**M1**	21225_2#178	Chicken	Vietnam
**M2**	09–05726	Human	Germany
**M3**	ZTA1601993EC	Chicken	Spain

To facilitate experimental strain detection in complex, non-sterile matrices by adding a selective culturing step, all 17 strains were artificially selected for rifampicin resistance. To this end, a 50 ml overnight culture of each strain was centrifuged at 4000 x g for 10 min, the pellet resuspended in 1 ml of LB media, and then plated on LB-agar plates containing 100 μg/ml rifampicin (Carl Roth, Karlsruhe, Germany). After overnight incubation at 37 °C, the plates were inspected for colony growth. The *rpoB* genes of the rifampicin-resistant strains were sequenced and one mutant was selected per experimental strain.

### ORFan gene identification

The DNA isolated from each strain was sequenced using Illumina short read technology (see Supplemental Table S1). The draft genomes were annotated by Prokka v1.13 [20] using a genus-specific blast for *E. coli*. The *pan*-genome was constructed at 95% amino acid identity by using Roary v3.12.0 [21]. Genes found in 99% of the strains within our collection were considered to represent core genes and the remaining genes classified as accessory genes. Paralogs were split into different orthologous groups. The strain-specific genes were identified by in-house scripts based on the binary matrix of gene presence or absence obtained from Roary. The nucleotide sequences of these strain-specific genes were extracted for each strain, and their specificity was further confirmed by using BLAST [22]. Each strain-specific gene was scanned against the entire gene pool of the HECTOR strain collection. Genes found in other strains or in more than one copy in the same strain at 90% identity and 90% coverage were discarded. A gene with a single copy present only in one strain was considered as strain-specific in this study. Its corresponding sequence was extracted based on the respective locus-tag. These genes were further examined individually via online BLAST analysis [23] against the GenBank [22] database specific for *E. coli* in order to assess exclusivity. As databases, the “Standard database” and the “Nucleotide collection (nr/nt)” were selected, and the organism was specified as “*Escherichia coli* (taxid: 562)”. As program selection, the search was optimized for “highly similar sequences (megablast)” and when no results were given, a new search was performed with the “somewhat similar sequence (blastn)” option selected. At the moment of the analysis (between February and June 2019), the GenBank database was composed of 4 representative *E. coli* genomes, more than 8000 complete *E. coli* genomes and more than 31,000,000 drafted *E. coli* genomes. If the top results from the BLAST [22] analysis of the GenBank [23] database belonged to known plasmids and fully matched the sequences being compared, they were considered plasmid-derived, and excluded from further analysis. If no hits were found from the search, a more exhaustive search against the non-redundant nucleotide database from GenBank was carried out. Those genes with few (< 10) or no hits were considered ORFans in this analysis.

### PCR and qPCR primer design

The ORFan genes detected in silico were used to design primers. If no “low-hit” ORFans were available for a specific strain, those regions within a selected ORFan gene, identified in another strain, but showing a higher sequence variation when blasted against the GenBank database were used to generate the primers. All PCR and qPCR primers were manually designed for each strain according to the following criteria: primer length and melting temperature, avoidance of dimers, hairpins, and self-complementarity. Specificity verification was performed using the tool Primer-BLAST [23]. The PCR primers were designed so that four multiplex PCR reactions could be performed in order to qualitatively (i.e., presence/absence from a given sample) trace all 17 experimental strains with a minimum number of reactions. For this, both melting temperatures of the primers and sizes of the products within a multiplex were selected so that the temperature should not differ more than 1 °C between primer pairs, and product sizes should differ by at least 100 bp from each other, if possible. The oligonucleotide primers were synthesized by Eurofins Genomics (Ebersberg, Germany). Their sequences are listed in Table 2.

**Table 2:** List of strain-specific PCR primers

Strain^**a**^	Gene locus tag	Primers		Ann. T° (°C)	Product (bp)
		Forward (5′ → 3′)	Reverse (5′ → 3′)		
**ChH1**	ECMDIDHM_04458	GGAAACGGATTACTTCTACG	CTGATAGAGATTCAGTCCCC	48	793
**ChH2**	ANLLMAEJ_01457	CCTACGCCACTAAACTACTG	CTATCATCACTGGAAATCCTG	47	560
**ChH3**	PDAKODAI_04433	GAATTACCGTCGTAGAGCAG	GGTATGGACTCAATGACAC	47	970
**C1**	KGJMKMMK_04916	CTGCTGTTATTAATCGGCTTGG	CTCTCAAGCGTGCTTTCTATC	51	886
**C2**	KGPFHGEA_00668	GTGGTGTAGAATTTATCGCATCC	AGCCATTCGATGAAACCAAG	51	665
**C3**	AMKFMELI_04046	GGGTCGATAACTTAGCAAGC	CTTGTTTGCAGAATGCTGCG	51	465
**C4**	LCFLNNEA_01211	GAATGGCAGGGCATACAAG	CAGACCTATGAACCTCTCCC	52	522
**C5**	GKDLAHGH_04328	CAGGAATGACAAACCTCTCG	CCGAACCATCGATTTGTCTG	51	1097
**C6**	LDHELLGP_02401	CTGCGGAAGAGTGTAAGTTC	CAGCGTCATCACTAAGCATT	51	162
**P1**	NIHDEHIB_01629	GTTAGCGGAACTCCAGCGGA	CCTCAGGTGCTTACTACGTTC	53	178
**P2**	EKBHOPAC_04494	CCAGATAGAGTCGTTTCTGC	GCAGCATTAACAGTAGGTCC	50	434
**P3**	DGMDDDKP_04627	GGCGATACGATTTTAACACCA	GCAACGGTCTAACATTCGCTG	50	347
**P4**	LLPKFPJB_0002	GATATAGCAAAAGCCGTTTCCTG	GCCTAGCAATAAATAACCGGTC	51	564
**P5**	HEMKEPCD_04830	GCTCGACATATTCCGAACAG	CACAGTTCTGGTGCAATAGAG	51	267
**M1**	PJHDGJGH_02587	GGCCATTGATAGCAGCATTG	CCGAATAATAACCATCGCC	48	371
**M2**	HDADFJHI_04570	CAGTTATGCTGGGCTAATTG	TGCGTAATTTGCATGATATGG	48	485
**M3**	EBAAKEFM_00440	CAGCAACGGATTGATACCTC	GCGAAGTTCTTCAATCTCC	48	697

^a^For specific strain information, see Table 1

Primers for qPCR were optimized for use with the Luna qPCR mastermix (New England Biolabs Inc., Ipswich, MA, USA). Amplicon sizes ranged between 100 and 250 bp, with a GC content from 40 to 60%, a melting temperature not greater than 61 °C with less than 1 °C difference between primers of the same pair, and a primer length of 19 to 25 nucleotides. If possible, ORFan gene and ORFan gene region used to design the qPCR primers were the same as the ones used for PCR primer design. The oligonucleotide primers were manufactured by Eurofins Genomics (Ebersberg, Germany). Their sequences are listed in Table 3.

**Table 3:** List of strain-specific qPCR primers

Strain^**a**^	Gene locus tag	Primers		Ann. T° (°C)	Product (bp)
		Forward (5′ → 3′)	Reverse (5′ → 3′)		
**ChH1**	ECMDIDHM_04458	CGCTACCAGGGACAGTACCT	TGATAGAGATTCAGTCCCCCCG	60	132
**ChH2**	ANLLMAEJ_01457	ACGAATGTGACCGAGCAGAG	CAGCGTACACCGAGTAAAACC	60	159
**ChH3**	PDAKODAI_04433	CGGAATTACCGTCGTAGAGCAG	CAGCACGATCACCAGAATAGAAGTG	60	115
**C1**	KGJMKMMK_04916	TGACAGCGAAAACCCAGCTC	CAGTTTGCCCCTGGATTTCC	60	151
**C2**	KGPFHGEA_00668	TGCGTTTGCAATTTACGGCG	GCGGCTCTATCCTTTGAGTCG	61	198
**C3**	AMKFMELI_04046	CAAACTCGACAAGAGCAACGC	AGAAGCAAAGAAACCGCCCC	61	198
**C4**	LCFLNNEA_01211	GCGAATTGCCAAAGAAAGCCAG	TGCGGATATGCAGCAAATCTCC	60	108
**C5**	GKDLAHGH_04328	GGCAGGGCCAAGCTTTAGTAC	GAGCTGCAAAACATGCCCATAC	61	173
**C6**	LDHELLGP_02401	GGCTGCGGAAGAGTGTAAGTT	GCGGCTCATATTTTTCATCAGCGTC	61	182
**P1**	NIHDEHIB_01629	CAACGAGTTAGCGGAACTCCA	CTTCTTGGCAATCAGCACAGC	60	161
**P2**	EKBHOPAC_04494	GAGTGGAGCCATGACTTCTGC	CCAAACGCCTAATATTTCTGCGACA	61	157
**P3**	DGMDDDKP_04627	CATGTCTTCTAATGGCGGTCGT	GTGCAACGGTCTAACATTCGCT	60	123
**P4**	LLPKFPJB_0002	CGCAGACTCTATTGCGTCTGG	GGACTTGCGATGTAGAATCCAATC	60	132
**P5**	HEMKEPCD_04830	AGCGGGCCGATGACAAATAC	TACAGGAAGCCGATAACCCCAC	61	184
**M1**	PJHDGJGH_02587	CTTGCGATTGAATCTGGCAGTGT	CACACCAGCATCTATTAAGCCCTG	60	168
**M2**	HDADFJHI_04570	CGACTGGCGCAATAACCAC	AGCACACCCGTCTTCATCATC	60	151
**M3**	EBAAKEFM_00440	CGGCACAGGCGGAAAAAAC	CGTCACCTCGTCTCCAAACATAAAG	61	250
**uidA**^**b**^	–	GCGAGGTACGGTAGGAGTTG	GAAGGGCGAACAGTTCCTGA	60	101

^a^For specific strain information, see Table 1

^b^Primers for the *E. coli* gene *uidA* (internal control)

### PCR multiplexes

Four multiplexes were needed in order to detect all 17 experimental strains. As much as possible, multiplexes targeted strains originating from the same host. In the case of the “Mix multiplex”, due either to fragment size or melting temperature three strains were included that didn’t fit in the other multiplexes. Multiplex PCR conditions were optimised following the recommendations published by Zangenberg et al. [24] or the PCR mastermix manufacturer. PCR was performed in a total volume of 25 μl containing 2 μl of purified DNA, 12.5 μl of OneTaq 2x Master Mix with Standard Buffer (New England Biolabs Inc., Ipswich, MA, USA), 9.5 μl of nuclease-free water, and 0.5 μl each of 10 μM forward and reverse primer. The reactions were performed in a Biometra T3 Thermocycler System (Analytik Jena, Jena, Germany) using the conditions specified in Table 4.

**Table 4:** PCR Multiplex description

Name of multiplex^**a**^	Strains detected^**b**^	Conditions		
		Step	Temp (°C)	Time (s)
**Chicken-Human**	ChH1	Initial denaturation	94	30
	ChH2	30 cycles	94	30
	ChH3		48	40
			68	60
		Final extension	68	300
**Cattle**	C1	Initial denaturation	94	30
	C2	30 cycles	94	30
	C3		52	60
	C4		68	70
	C5	Final extension	68	300
	C6			
**Pig**	P1	Initial denaturation	94	30
	P2	30 cycles	94	30
	P3		51	35
	P4		68	40
	P5	Final extension	68	300
**Mix**	M1	Initial denaturation	94	30
	M2	30 cycles	94	30
	M3		49	40
			68	45
		Final extension	68	300

^a^From now on, referred to as Chicken-Human multiplex, Cattle multiplex, Pig multiplex and Mix multiplex in the text

^b^Strain information displayed in detail in Table 1

Gel electrophoresis was performed by using 1.0% all-purpose, high-purity agarose (VWR International, Radnor, PA, USA) gels with 0.25X SERVA DNA stain clear G (SERVA Electrophoresis GmbH, Heidelberg, Germany) in 1X Tris-borate-EDTA buffer (VWR International, Radnor, PA, USA) in a Perfect Blue Gel System (VWR International, Radnor, PA, USA) at 150 V for 1 h. Two microliters of amplified DNA were mixed with 4 μl of gel loading dye (New England Biolabs Inc., Ipswich, MA, USA) for analysis. For reference, a Quick-Load Purple 100 bp DNA Ladder (New England Biolabs Inc., Ipswich, MA, USA) was used (bands every 100 bp up to 1000 bp, plus two additional bands at 1200 bp and 1500 bp).

### Quantitative PCR reaction setup

Quantitative PCR conditions were optimised following the qPCR mastermix manufacturer’s specifications. Assays for qPCR were performed on a CFX96 Touch Real-Time PCR Detection System (Bio-Rad Laboratories, Hercules, CA, USA). Reactions contained a total volume of 20 μl, in which 2 μl of purified DNA were used together with 10 μl of Luna Universal qPCR Master Mix (New England Biolabs Inc., Ipswich, MA, USA), 7 μl of nuclease-free water, and 0.5 μl each of 10 μM forward and reverse primer. Each reaction was performed in triplicate. The cycling conditions included an initial denaturation step of 1 min at 95 °C followed by 40 cycles of 95 °C for 15 s and 60 °C for 30 s. No-template controls (2 μl of nuclease-free water instead of DNA extract) and an internal calibrator control for each strain (2 μl of each strain’s purified DNA at a concentration of 10 ng/μl with a known Ct value, ranging between 12 and 14 Ct, used to account for possible variations between plate runs) were performed with each batch of samples tested. The *uidA* gene encoding a β-glucuronidase specific for *E. coli* was included in the qPCR assay as housekeeping gene [25].

### Specificity and efficiency testing

Primer pairs were individually tested with their respective target strain first by simplex PCR and afterwards together in multiplexes. For this, each strain was streaked onto a Gassner agar plate (Sifin, Berlin, Germany) containing ceftiofur at 4 μg/ml (ceftiofur hydrochloride, VETRANAL®, St. Louis, MO, USA) and allowed to grow overnight at 37 °C. After verifying a pure culture, a single colony of each strain was then picked and used to inoculate 10 ml of fresh LB medium. Liquid cultures were incubated overnight at 37 °C. After 18 h, 3 ml of the overnight culture were used to isolate genomic DNA (peqGOLD Bacterial DNA Mini Kit, Peqlab, Erlangen, Germany). A Nanodrop microvolume spectrophotometer (NanoDrop One, Thermo Fisher) was used to monitor DNA quality and concentration. Serial dilutions (100 ng/μl to 0.1 ng/μl) were made from the DNA solutions and used as templates to test primer specificity. Culture mixtures of different strains were also used to ensure no cross-detection occurred. Faecal spiking was performed to verify that the primers were sufficiently specific to detect individual strains in the background of an intestinal microbiome. For this, pure cultures of each strain were allowed to grow overnight at 37 °C in liquid culture. A hundred microliters of the overnight culture were used to inoculate 10 millilitres of fresh media. When the culture reached an OD_600_ of 0.5, each individual culture was adjusted to a bacterial concentration of 5.88 × 10^8^ cells, based on previously collected data, and 10 microliters of the concentration-adjusted culture were used to spike 1 g of a porcine faecal sample. Single-strain spiking and spiking with mixtures containing 2–10 of the experimental bacterial strains were performed. Afterwards, DNA was extracted using the Quick-DNA Faecal/Soil Microbe Miniprep Kit (Zymo Research, Irvine, CA, USA).

The efficiency of the qPCR primers was calculated following the recommendations published by Svec et al. [26]. Ten-fold dilutions ranging from 10 ng down to 0.0001 ng of DNA were tested in triplicate. The mean average of the triplicates was plotted on a logarithmic scale along with the corresponding template concentrations. A linear regression curve was applied to the data points, and the slope of the trend line was calculated. Finally, efficiency was calculated by using the equation: E = − 1 + 10^(− 1/slope)^. All primer pairs tested showed efficiency values between 90 and 95%. Non-template controls did not show any amplification, and internal calibrator values always stayed within the determined Ct value range of 12–14 cycles depending on the respective calibrator used.

### Tracing of bacterial strains after cocktail infection of piglets

For in vivo analysis, pigs were inoculated with a bacterial cocktail containing 17 different strains (Table 1). The animal experiment was approved by the competent authority (State Office for Agriculture, Food Safety and Fisheries of Mecklenburg-Western Pomerania, Rostock, Germany, reference no. 7221.3–1-034/19). Eight German landrace pigs, 42–45 days old and healthy as per veterinary guidelines, were purchased from a conventional pig farm (bhzp Garlitz, Langenheide, Germany) and housed in an environmentally controlled animal facility at the Friedrich-Loeffler-Institut (FLI) on the Isle of Riems, Greifswald. The animals adapted to the environmental conditions for 3 weeks prior to experimental infection. Meanwhile, faecal samples were collected to determine the resistance status of the coliform bacterial population in the intestinal tract of the pigs. Some samples tested positive for ceftiofur-resistance, but all samples tested negative for ceftiofur/rifampicin double-resistant bacteria. All inoculation strains were grown individually on Gassner plates containing 4 μg/ml ceftiofur and 50 μl/ml rifampicin and stored at 4 °C. The bacterial cocktail was prepared before inoculation, by mixing liquid cultures of all 17 strains at equal numbers (5.88 × 10^8^ cells per strain) in order to reach a total of 10^10^ bacteria per inoculation dose. Mixtures were gently centrifuged, the media removed, and the bacterial pellets resuspended in 10 ml of physiological saline containing 10% sodium bicarbonate to buffer stomach acid. After re-suspension, cooled individual doses were immediately transported to the animal facility. For inoculation, all animals were lightly sedated intramuscularly with azaperon (Stresnil®, Elanco, Greenfield, IN, USA) using 0.5 ml / 20 kg of body weight. The inoculation of the strain cocktail was performed intra-gastrically using a gastric tube (B. Braun, Melsungen, Germany). The animals recovered quickly and were fed immediately after the procedure. Post-inoculation, clinical observation of the animals was performed once per day during the entire experiment. Rectal swabs, in addition to faecal samples from the pen, were collected daily from day 1 to 14 post-infection, and every 2 days from day 15 until day 56 at the end of the experiment.

Rectal swabs were suspended in 1 ml of LB medium and allowed to rest at 37 °C for 30 min. The swab wash-offs were serially diluted from 10^− 1^ to 10^− 4^ and used for plate spotting on Gassner agar plates containing either no antibiotics or ceftiofur (4 μg/ml) and rifampicin (50 μg/ml). For spotting, 10 μl droplets of the 10^− 1^ to 10^− 4^ dilutions were gently spread on each plate in duplicate and plates were left open inside the bench for 1–2 min to allow excess media to be absorbed by the agar. After overnight aerobic incubation at 37 °C, colonies were counted in each droplet. In addition, 100 μl of the suspension from the rectal swabs were plated on Gassner agar plates containing ceftiofur (4 μg/ml) and rifampicin (50 μg/ml). After overnight incubation at 37 °C, plates were washed off using 2 ml of LB, and the suspension was used to isolate DNA with a commercial kit (peqGOLD Bacterial DNA Mini Kit, Peqlab, Erlangen, Germany).

To collect colonic content and tissue, four animals each were euthanized by intravenous administration of Pentobarbital (Release® 500 mg/ml, WDT) on days 43 p.i. and 56 p.i., respectively. At *post-mortem* examination, the intestinal tract of each animal was removed and the colon section was separated by a double ligation. After opening, approximately 50 ml of the content was collected and a large piece of intestinal tissue (approximately 2–3 cm) sampled and gently washed to remove any remaining content.

One gram of content was weighed, diluted in 9 ml of LB medium and allowed to rest at 37 °C for 30 min. One gram of tissue was weighed, finely chopped, suspended in 9 ml of LB medium and allowed to rest at 37 °C for 30 min. One hundred microliters of each suspension were serially diluted from 10^− 1^ to 10^− 4^ and used for plate spotting (see detailed description above) on Gassner agar plates containing either no antibiotics or ceftiofur (4 μg/ml) and rifampicin (50 μg/ml). After overnight incubation at 37 °C, colonies were counted.

One millilitre of the initial dilution (10^-1^) was used to isolate DNA with a commercial kit (peqGOLD Bacterial DNA Mini Kit, Peqlab, Erlangen, Germany) prior to enrichment.

Sample enrichment was performed by adding rifampicin to the initial content suspensions, which was further incubated overnight at 37 °C. The next day, 100 μl of the overnight suspension were plated on Gassner agar plates containing ceftiofur (4 μg/ml) and rifampicin (50 μg/ml). After overnight aerobic incubation at 37 °C, plates were washed off using 2 ml of LB. One millilitre of the suspension was used for generating glycerol stocks and the remaining millilitre was used to isolate DNA with a commercial kit (peqGOLD Bacterial DNA Mini Kit, Peqlab, Erlangen, Germany).

### Statistical analysis

Statistical analyses were performed with GraphPad Prism Software (GraphPad Prism version 9.0.2 for Windows, GraphPad Software, San Diego, California USA, www.graphpad.com). The one-way ANOVA on ranks (Kruskal-Wallis) test for multiple comparisons was used to determine the significance of differences between ∆Ct (∆Ct = Ct (gene of interest) – Ct (housekeeping gene)) values from strains. Values with *p* ≤ 0.05 were considered significant.

## Results

### ORFan gene identification

A total of 299 ORFan genes were identified in the whole genome sequences of the 17 experimental strains (Table 5).

**Table 5:** List of ORFan genes identified per strain

Strain	N° strain-specific genes	N° of remaining ORFan genes	N° genes classified as putative plasmid-encoded
**P3**	158	85	71
**ChH3**	36	29	7
**P2**	14	13	0
**ChH2**	13	13	0
**C3**	13	13	0
**M3**	13	11	2
**P5**	11	11	0
**M1**	11	9	0
**P4**	10	8	0
**C5**	6	2	4
**C4**	5	5	0
**ChH1**	3	3	0
**C2**	3	3	0
**P1**	2	1	1
**C1**	1	0	1
**C6**	0	0	0
**M2**	0	0	0

The number of strain-specific genes per strain was highly variable, ranging from a maximum of 158 in one strain to zero in two strains. Approximately one-third (86) of the 299 strain-specific genes were classified as plasmid-borne and, therefore, not deemed suitable for strain identification. They were not further analysed as to their ORFan status. Nearly all of the strain-specific genes, that were not potentially plasmid-encoded (213), turned out to be ORFan genes (206). For the three remaining strains that showed no ORFan genes, a comparison within the 17 strains selected for the bacterial cocktail was made. Genes that were unique among the cocktail strains, and showed less than 5 hits with other *E. coli* in the GenBank search, were then used as strain identifiers. These primers were tested with DNA isolated from faecal samples from clinically healthy (non-inoculated) pigs with no positive matches, and were deemed specific enough to be used during the animal experiment.

### PCR primer specificity and multiplex functionality

**Fig. 1:**
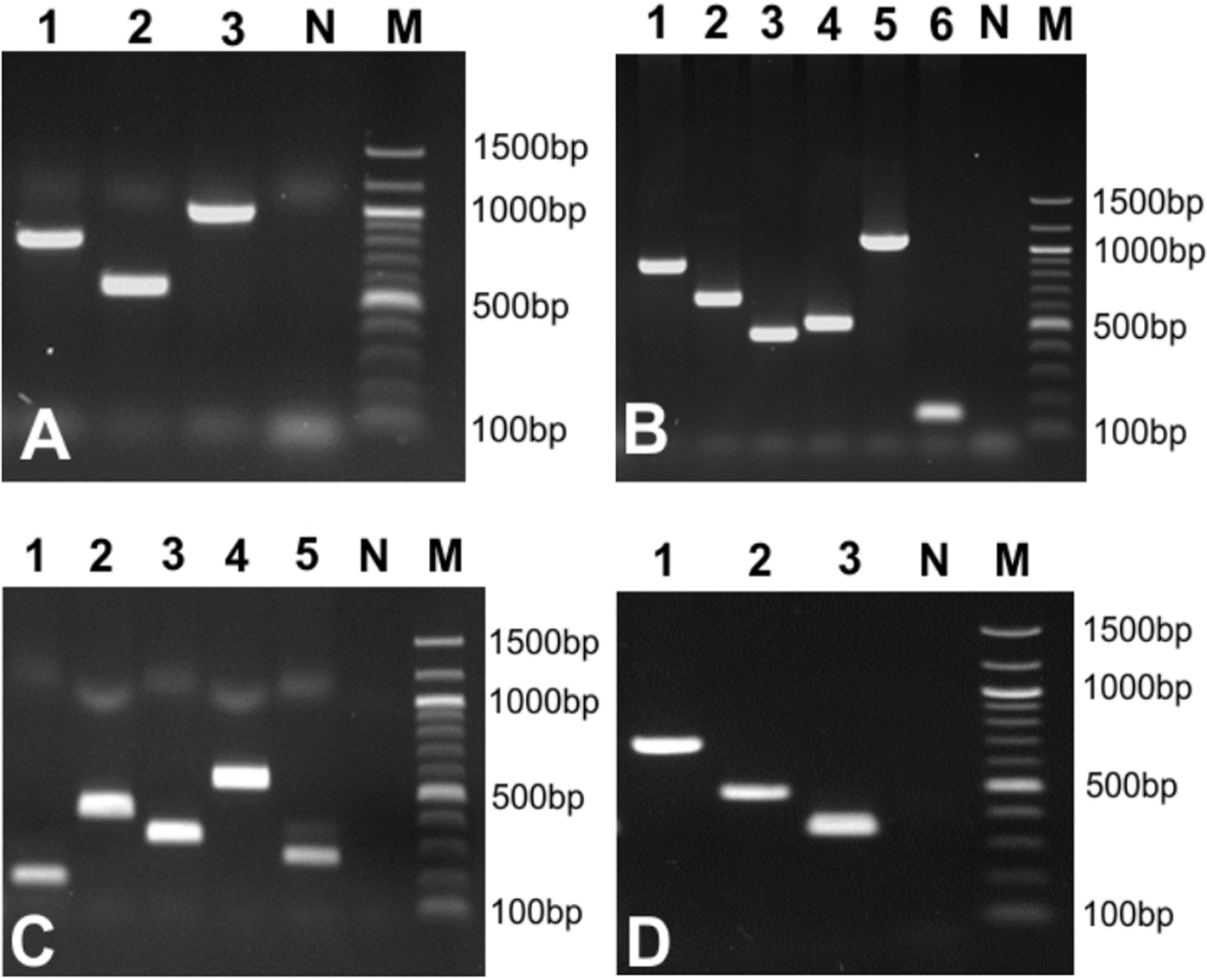
Results of the multiplexes designed and their specificity for the target strains. DNA was isolated from each bacterial strain and tested with each of its corresponding multiplexes. Gel **A** – Chicken-Human multiplex: strains ChH1 (1), ChH2 (2), ChH3 (3); Gel **B** – Cattle multiplex: C1 (1), C2 (2), C3 (3), C4 (4), C5 (5), C6 (6); Gel **C** – Pig multiplex: strains P1 (1), P2 (2), P3 (3), P4 (4), P5 (5); Gel **D** – Mix multiplex strains M1 (1), M2 (2), M3 (3). Lane N: Negative control; lane M: 100-bp marker (New England Biolabs Inc., Ipswich, MA, USA)

A primer pair was designed for each experimental strain (Table 2). Only primer pairs that exclusively amplified DNA of the corresponding strain were considered target-specific. Among the 34 initially designed primers, seven pairs failed (41.2%), either by not-amplifying their respective target or by yielding non-specific bands when tested against other strains. Consequently, seven new primers pairs were designed and two pairs again failed (28.5%) for the same reasons. The third pair of primers yielded specific signals for the last two strains. Combining the primer pairs into multiplexes affected neither their specificity nor their ability to identify the matching strain (Fig. 1). No non-specific bands were seen after multiplexing the primers; however, it was observed that the bands of the smallest fragments in the multiplex (smaller than 150 bp) had reduced intensity in those multiplexes containing a larger number of primer pairs (e.g., Cattle multiplex with six pairs of primers and Pig multiplex with five pairs of primers – see Table 4).

During multiplex testing with DNA isolated from pure cultures, the detection limit of the reactions ranged between 1 to 10 ng/μl DNA, which is equivalent to 200 to 2000 genome copies. However, DNA isolated directly from faecal samples spiked with 10^8^ bacteria did not yield positive results, indicating that inhibitory substances or the high endogenous background of bacterial and eukaryotic DNA might be problematic in the aforementioned set up. To overcome this obstacle, a selection step was added for experimental strain detection from faecal samples. Since all experimental strains possessed ESBL genes and were rifampicin resistant, a plating step on Gassner agar containing ceftiofur (4 μg/ml) and rifampicin (50 μg/ml) was added to limit strain isolation to the experimental strains and to eliminate the presence of inhibitory faecal substances. This eliminated growth of other endogenous ESBL bacteria that were already present in the animals pre-inoculation, and facilitated specific detection of experimental strains in a multiplex PCR approach using boiled lysates or DNA prepared from the pooled bacteria.

### Monitoring of faecal samples

**Fig. 2:**
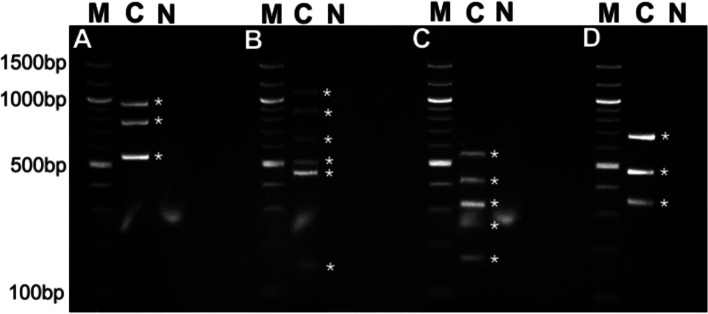
Results of the four multiplexes after testing DNA isolated from the inoculation cocktail. The PCR multiplexes showed that all 17 strains were present in the cocktail before animal inoculation. Lane M: 100-bp marker; lane C: DNA isolated from the experimental cocktail; lane N: negative control. Gels: **A** - Chicken-Human multiplex; **B** - Cattle multiplex; **C** - Pig multiplex; **D** - Mix multiplex. Asterisks (*) denote strain-specific PCR signals

The suitability of the ORFan approach to qualitatively monitor shedding of different *E. coli* strains by pigs when inoculated with 17 strains simultaneously was assessed by conducting colony counting of resistant *E. coli* and detection of strains by classical PCR. All experimental strains were confirmed to be present in the initial inoculation cocktail prepared to be given to the animals (Fig. 2). After inoculation of the animals with the bacterial cocktail, faecal samples were collected for 56 days. Results after the first 24 h post-inoculation were highly variable between animals. After 48 h, 12 of the 17 strains were detected in at least one animal, with a minimum of four strains and a maximum of 12 strains detected in the eight animals. The remaining five strains were not detected at all in faecal matter during the experiment.

**Fig. 3:**
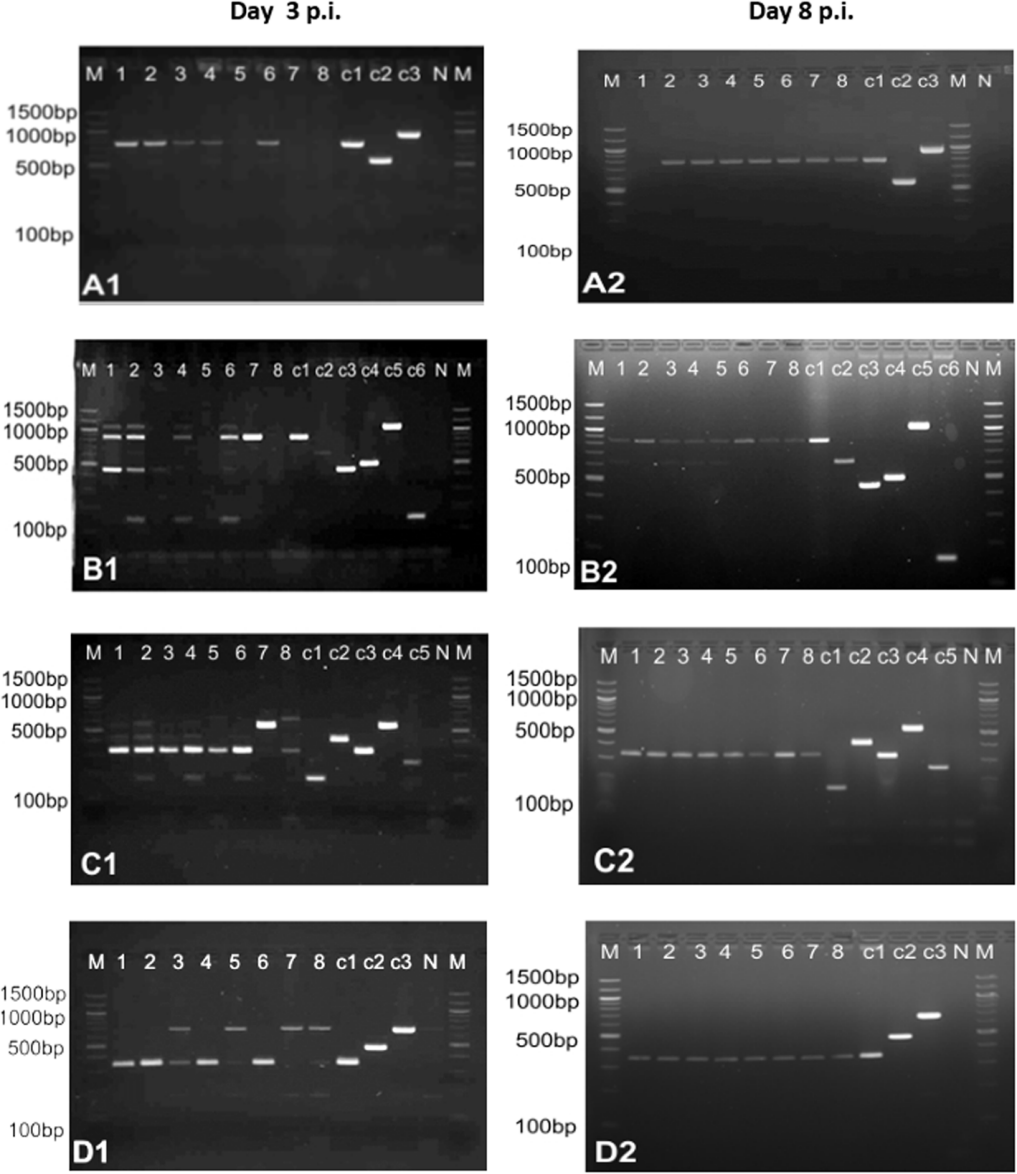
Results of the four multiplexes after testing DNA isolated from faecal samples 3d p.i. **A1-D1** and faecal samples 8d p.i. **A2-D2** Lane M: 100-bp marker, N: Negative control, c1-c6: positive controls for each individual multiplex (controls shown in Table 1), lanes 1–8: samples isolated from each of the eight experimental pigs. Gels: **A** – Chicken-Human multiplex; **B** – Cattle multiplex; **C** – Pig multiplex; **D** – Mix multiplex

Experimental strain counts, i.e., the total number of colonies, which grew without differentiating the single strains, remained high up to day 3–4 post-inoculation, after which they slowly declined. A significant number of strains from the inoculum mixture were detectable at day 3 (Fig. 3), with three animals being positive for four strains, one animal for six strains, one animal for nine strains, two animals for eleven strains, and one animal positive for twelve strains. Inoculated bacteria were shed and identified by PCR up to day 21 p.i., when their counts on selective plates had declined to single-digit numbers per gram of faeces or were absent, requiring an enrichment protocol for further detection of experimental strains. By day 29 p.i., only 5–6 different strains were shed, even after enrichment, and by day 53 p.i., only four experimental strains were detected.

### qPCR system results

**Fig. 4:**
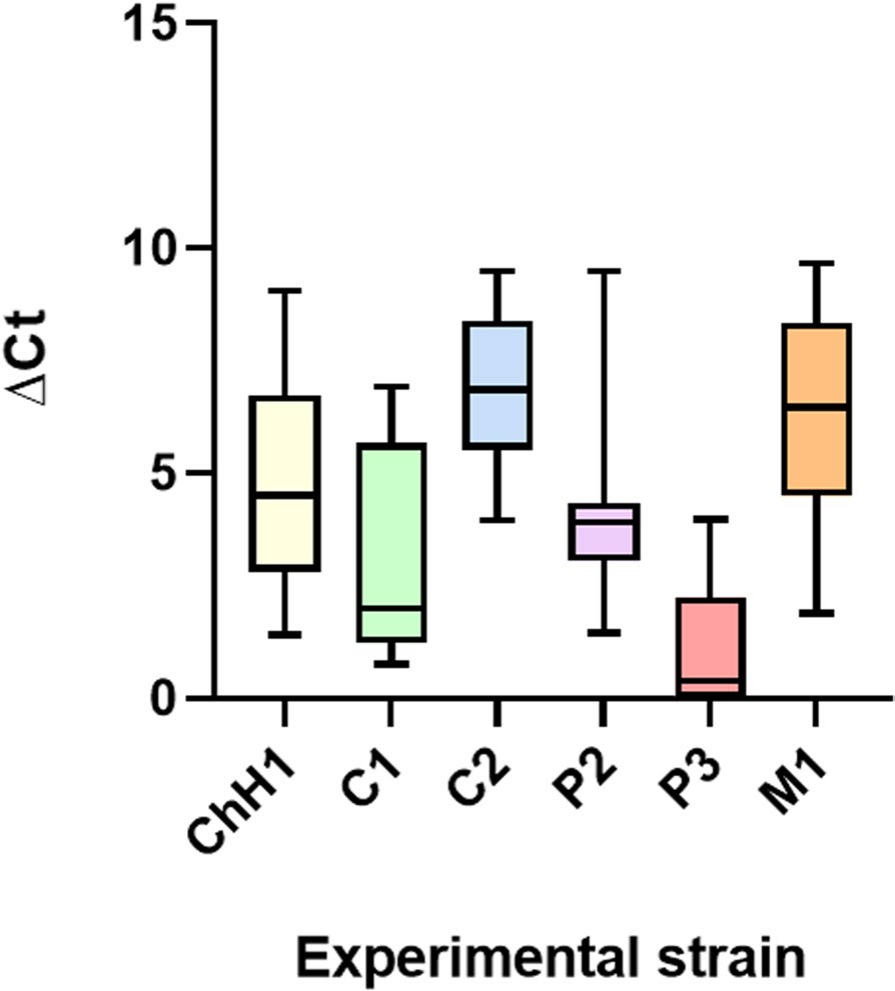
Presence of six different experimental strains in samples taken at necropsy from the large intestine of pigs, which had been orally inoculated with a mixture of 17 *E. coli* strains 56 days earlier. ΔCt values obtained with samples from individual pigs are depicted on the y-axis (geometric mean and quartiles for all animals; *n* = 8). Specific *p*-values for the differences in quantitative values for detecting individual strains are given in Table 6

The qPCR system was used to prove the general suitability of the ORFan approach to compare different *E. coli* strains quantitatively within complex intestinal porcine microbiomes. To this end, strains that gave positive signals in the PCR Multiplex setup were further analysed via qPCR for their presence in the dissection sample contents. Delta-Ct (ΔCt) values obtained from the qPCRs showed significant differences in quantities between the strains. The strain classified as P3 (39533) showed the lowest ΔCt values, indicating a high presence in the colonic content. This strain’s presence in the samples was significantly different from all other strains tested via qPCR (Fig. 4; Table 6). The strains classified as C2 (R45) and M1 (21225_2#178) showed the highest ΔCt values on average, denoting both strains’ lower presence in the intestinal content of the inoculated animals. Also, M1, together with P2 (IMT28138), showed the lowest degree of significance when compared against the other strains tested (Fig. 4; Table 6).

**Table 6:**
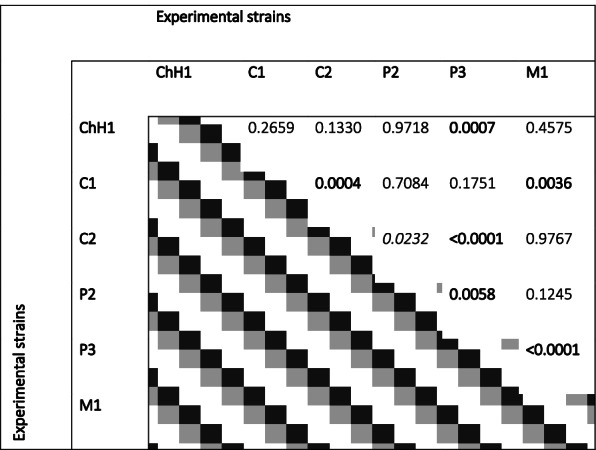
List of *p*-values when comparing ΔCt values for detection of experimental strains present in large intestinal content of inoculated pigs^1^

^1^The *p-values* < 0.05 were considered significant (italics), *p-values* < 0.01 were considered highly significant (bold)

## Discussion

Current strain identification methods based on DNA fragment patterns, including pulsed-field gel electrophoresis (PFGE), restriction fragment length polymorphisms (RFLP), repetitive sequencing-based PCR (REP-PCR), Enterobacterial Repetitive Intergenic Consensus PCR (ERIC-PCR), and multiple-locus variable-number tandem repeat analysis (MLVA) [3], cannot be used to characterize mixtures of strains because it is not possible to assign individual bands to their cognate isolate unambiguously. DNA sequencing-based methods cannot resolve individual strains in a mixture except if sequences with differentiating SNPs have been identified for all strains. The analysis would then require prior testing of the animals to ensure the absence of these discriminatory SNPs in the endogenous bacterial population. DNA hybridization-based methods, such as cDNA and oligonucleotide microarrays, permit the detection of individual genes or gene fragments and, consequently, strains, but they also require previous knowledge of strain-specific sequences and have to be individually adapted for each new combination of to-be-detected strains. Other strategies, commonly used in infection models, like introducing artificial selection markers, such as antimicrobial resistance genes or genes coding for fluorescent proteins, are of limited feasibility since they allow only a limited number of strains to be introduced simultaneously [27].

The ORFan gene targeting approach utilized herein has the advantage of allowing the introduction of multiple strains simultaneously into an experimental setup, including an in vivo animal trial, as demonstrated. As opposed to existing methods for strain identification, which are either extremely time-consuming, expensive or need specific equipment, the ORFan identification system can be implemented relatively fast and is accessible to everyone with standard laboratory equipment and moderate knowledge of bioinformatics. This method can also be flexibly and easily scaled up, because it only requires the identification of a specific ORFan gene for any novel strain to be introduced.

The ORFan gene approach allows the combination with other visualization techniques to expand strain-specific detection to other types of biological sample. Recently, a HiPR-FISH (high phylogenetic resolution - fluorescence in situ hybridization)-based identification technique was described [28]. In the aforementioned study, HiPR-FISH was employed to identify (i) over 1000 *E. coli* isolates, using artificially introduced barcoded sequences to generate fluorescent probes for individual strain identification and (ii) bacterial genera present in the murine gut microbiome or in human plaque biofilms using 16S rRNA sequences to identify different genera [28]. Using ORFan genes instead of 16S rDNA as targets for such strain-specific fluorescent hybridization probes could generate an alternative and complementary data set on individual strains. Fluorescent detection of ORFan gene presence via hybridization of fixed and permeabilized cells would allow rapid quantification of large samples via fluorescence-activated cell sorting. Another possible application could be specific strain detection in tissue sections from infected animals, which would even permit comparative spatial-temporal resolution of multiple strains in a host coupled with respective niche identification.

The ORFan gene identification workflow pinpointed a large enough number of ORFan genes for most strains to allow the successful selection of specific primers. Previous *E. coli* phylogenetic studies unveiled that ORFan genes could compose approximately 1% of the bacteria’s total core genome, with an increase of approximately 26 genes per new genome sequenced [29]. The average number of 18 specific genes per strain was lower for the set of strains selected here for the animal test, and the respective total number of strain-specific genes was lower for 15 of the 17 strains. An additional complication is that these genes could also be located on plasmids and, thus, subjected to horizontal transmission, disqualifying them as stable markers. In our study, approximately one-third of the strain-specific genes were classified as plasmid-derived, but all affected strains, except one, harboured alternate ORFans for which a specific detection system could be designed. Primer implementation was an iterative process, requiring up to three rounds of sequence selection until all 17 strains could be explicitly identified in multiplex PCR assays. Each round required up to 2 weeks of time spent between ORFan gene selection and successful testing of the specific primer pairs. For the three strains, for which no ORFan genes were identified, an additional search of their accessory genomes was performed to identify unique genes within the sequence context of the 17 experimental strains. ORFan genes found using this approach were also subjected to the workflow presented in the “Materials and Methods” section to assess exclusivity. As expected, most of the newly identified genes did have more than 10 hits in the GenBank search. For this reason, only nucleotide stretches within a selected ORFan gene that showed a higher degree of sequence variation between the experimental strain’s sequence and the sequences available in GenBank, were used to generate primers. Furthermore, designated cocktail strains were artificially rendered rifampicin-resistant to distinguish them from cephalosporin-resistant *E. coli* in the endogenous animal microbiota. The combination of these measures was successful for expanding the limits of the ORFan approach, at least for the list of bacteria under study and in the group of animals used.

An aliquot from the strain cocktail used to infect the animals was immediately stored at −80 °C after preparation. DNA was extracted, and the four multiplexes performed to corroborate the experimental strains’ presence. All 17 strains were detected, confirming their presence in the inoculation cocktail. Their respective band intensities were also similar, indicating that each strain had been added in a similar quantity to the cocktail. Among the 17 strains used to infect the pigs, five strains were not detected in the faecal samples throughout the experiment and were also not detected in the intestinal content at necropsy. Eight strains were only detectable intermittently during the entire experiment, with all of them displaying higher prevalence during the first 14 days p.i.. The remaining four strains were consistently detected throughout the entire experiment. The presence or absence of the 12 successfully detected strains was closely monitored during the entire experiment via the multiplex PCRs, demonstrating that the detection system can be used to follow dynamic changes in a strain’s presence.

All qPCR primers tested showed high efficiency with values ranging from 90 to 95%. Several tests with various samples corroborated that the primers were indeed sufficiently specific to allow the direct use of faecal DNA to detect experimental bacteria from the mixed background of total bacteria. Strain presence in gut content from the animals’ colon, which had already been demonstrated in the multiplex PCRs, was also seen with qPCR primers. The qPCR data indicated colonization differences between experimental strains. Based on the qPCR results, strains with high colonization capacity were also easily re-isolated from the faecal samples, demonstrating that the qPCR results are accurate at monitoring specific strain abundancies in the colonic samples.

Next-generation metagenome sequencing provides vital information on microbial populations and genetic diversity at all taxonomic levels. A fast and easy but robust and reliable method for individual strain identification based on information derived from whole-genome sequencing has yet to be described. The usage of ORFan genes, specific for individual strains, could be such a valuable tool, as it allows to develop precise PCR markers for tracing the strains in complex mixed-culture experiments. The method has the potential to be applied in multiple ways to foster our understanding of, for example, the population dynamics of closely related strains of a pathogen. A probiotic or any other strain of interest can be evaluated as to its colonization ability, general strain fitness, or zoonotic risk and might guide the development of intervention strategies [3]. This method could also potentially be used as a fast and powerful tool for back-tracking the identification of specific pathogens in the event of an outbreak. At present, whole-genome-sequencing is an essential part of outbreak investigations. After acquiring the sequence data of the suspected outbreak agent, the strain detection method presented here could be used for rapid identification of a specific outbreak strain following identification of ORFan genes unique to the specific outbreak agent and the design of strain-specific primers. Without the need to amplify a whole set of virulence markers characteristic of an outbreak strain or to isolate the pathogen from multiple samples, ORFan genes may be used for specific-strain identification via PCR, allowing the rapid pre-screening of many different samples to narrow down the potential sources that could have served as origin or as potential transmission route of the pathogen in the outbreak. In the ensuing second step of outbreak analysis, PCR-positive samples would be subjected to classical approaches involving strain isolation and characterization for unambiguous identification of the culprit strain. This would allow to perform large-scale surveys of many different samples to identify potential outbreak sources and clusters. Similar approaches have been used in the past, such as the one described by Bielaszewska et al. [30], where an outbreak strain was successfully identified by detecting a specific virulence gene profile. A similar approach was described by Kiel et al. [31], where two pipelines were simultaneously run, comparing Shiga-toxin expressing *E. coli* (STEC) genomes versus control genomes and an STEC core proteome versus control proteomes. Lineage- and serotype-specific genes were identified this way and used for monitoring specific STEC strains.

## Conclusion

The method described in this manuscript using single unique genes to identify specific strains proved easy to implement and very reliable in identifying and following individual strains and their dynamics in an in vivo animal model of experimental infection, thus representing a fast, inexpensive and reliable alternative to more costly and laborious identification methods.

## Supplementary Information

**Additional file 1: Supplemental Table S1.** Isolate and sequencing metadata for the strains used in the study.

## Data Availability

The datasets generated and/or analysed during the current study are available in the NCBI repository, under the title “The impact of Host restriction of *Escherichia coli* on Transmission dynamics and spread of antimicrobial Resistance”, BioProject number PRJNA739205.

## Abbreviations

∆Ct: Delta threshold cycle
BLAST: Basic Local Alignment Search Tool
bp: Base pair
Ct: Threshold cycle
EDTA: Ethylenediaminetetraacetic acid
ERIC-PCR: Enterobacterial Repetitive Intergenic Consensus PCR
ESBL: Extended-spectrum beta-lactamase
FISH: Fluorescence in situ hybridization
FLI: Friedrich-Loeffler-Institut
LB: Lysogeny broth
MLVA: Multiple-locus variable-number tandem repeat analysis
ORFan: Open reading frames
PCR: Polymerase chain reaction
p.i.: Post-inoculation
PFGE: Pulsed-field gel electrophoresis
qPCR: Quantitative polymerase chain reaction
REP-PCR: Repetitive sequencing-based PCR
RFLP: Restriction fragment length polymorphisms
SNP: Single-nucleotide polymorphism
STEC: Shiga-toxin expressing *E. coli*
